# Driving-Induced Neurological Biomarkers in an Advanced Driver-Assistance System

**DOI:** 10.3390/s21216985

**Published:** 2021-10-21

**Authors:** Iqram Hussain, Seo Young, Se-Jin Park

**Affiliations:** 1 Center for Medical Convergence Metrology, Korea Research Institute of Standards and Science, Daejeon 34113, Korea; iqram@ust.ac.kr (I.H.); young2da@kriss.re.kr (S.Y.); 2 Department of Medical Physics, University of Science & Technology, Daejeon 34113, Korea; 3 AI-Based Healthcare Research Group, Sewon Intelligence, Ltd., Seoul 04512, Korea

**Keywords:** electroencephalogram, physiological biomarker, advanced driver assistance system (ADAS), mental workload, driving simulator

## Abstract

Physiological signals are immediate and sensitive to neurological changes resulting from the mental workload induced by various driving environments and are considered a quantifying tool for understanding the association between neurological outcomes and driving cognitive workloads. Neurological assessment, outside of a highly-equipped clinical setting, requires an ambulatory electroencephalography (EEG) headset. This study aimed to quantify neurological biomarkers during a resting state and two different scenarios of driving states in a virtual driving environment. We investigated the neurological responses of seventeen healthy male drivers. EEG data were measured in an initial resting state, city-roadways driving state, and expressway driving state using a portable EEG headset in a driving simulator. During the experiment, the participants drove while experiencing cognitive workloads due to various driving environments, such as road traffic conditions, lane changes of surrounding vehicles, the speed limit, etc. The power of the beta and gamma bands decreased, and the power of the delta waves, theta, and frontal theta asymmetry increased in the driving state relative to the resting state. Delta-alpha ratio (DAR) and delta-theta ratio (DTR) showed a strong correlation with a resting state, city-roadways driving state, and expressway driving state. Binary machine-learning (ML) classification models showed a near-perfect accuracy between the resting state and driving state. Moderate classification performances were observed between the resting state, city-roadways state, and expressway state in multi-class classification. An EEG-based neurological state prediction approach may be utilized in an advanced driver-assistance system (ADAS).

## 1. Introduction

Car driving demands low-level physical activities and heavy mental workloads, to deal with complex driving environments. Driving is a complicated attention-intensive and cognitively demanding task, and involves driving skills, understanding road scenarios, and drivers’ behavior [1]. The increased cognitive demand on the brain gives the drivers fatigue and subsequently drowsiness and boredom. The increased cognitive load is a significant source of road accidents and fatalities, which demands extensive studies. Modern vehicles are equipped with various luxurious components and driving information systems, such as navigation systems, on-board lifestyle accessories, personal communication devices, and music systems, which contribute to mental distraction [2]. Road traffic systems, including traffic signals, multi-lane roads, and information boards, and road conditions, such as road curvature and road slopes, affect the cognitive workload of the driver.

A drivers’ cognitive load can be tracked through several measures: physiological signals, vehicle driving data, and driving behavioral measures [3,4]. Tracking physiological signals is one of the essential methods for measuring cognitive outcomes. Real-time physiological monitoring can be employed for disease prognostics and functional response during regular activities, such as driving, walking, sleeping, and so on [5,6,7,8]. Physiological sensor data can be utilized for understanding neurological and cardiac stresses and delivering feedback to driver-assistance systems for safe-driving recommendations [9,10]. Vehicle driving data are one of the vital tools for understanding driving patterns and vehicle operational performance. Behavioral data can be accessed through cameras, motion sensors, and radar-based sensors to identify the responses of the driver to the driving scenario [11]. As the mental workload in a virtual driving environment parallels on-road driving, driving simulators have been successfully utilized in physiological and behavioral studies of driving [12,13].

Tracking brainwaves is one of the essential methods for assessing cognitive load, and electroencephalography (EEG) is the physiological tool for measuring the electrical potential from the scalp and that directly reflects the activities originated by the brain [14,15,16,17,18]. The cognitive and neurological workload of a driver can be recorded and analyzed using EEG. Studies showed that EEG event-related potentials (ERPs) metrics were associated with driving performance, and reduced ERP amplitude over the frontal lobe was associated with bad driving performance in a virtual car driving scenario [19]. Other studies utilized EEG signals to measure a driver’s mental workload with respect to various traffic and road conditions and to evaluate neurophysiological measures for providing insights about mental states while dealing with driving tasks of varying levels of difficulty [20]. Along with vehicle driving data, EEG was utilized to distinguish drivers’ mental workload, between ‘normal’ and ‘overload’ [21]. Driving behavior was investigated using EEG data to classify driving patterns with varying levels of stability and aggressiveness in a simulated car-following test [22,23]. EEG is also considered a tool for health monitoring and disease prognostics during driving [5,8]. Another study adopted EEG power spectrum features for driving state prediction in car-following experiments [24]. Several studies have investigated drivers’ fatigue through brainwaves and other physiological signals [25,26,27].

Most physiological studies of drivers’ mental states deal with a statistical investigation of the driving pattern, using frequency spectral components and ERP. The road scenario parameters, such as road type, the intensity of vehicles, the surrounding scenario, and the speed limit, are considered to affect the driving behavior and the neurological states of the driver significantly. A machine learning-based approach to characterize the EEG spectrum with various driving scenarios has not yet been explored.

Advanced driver-assistance systems (ADAS), a core technology in emerging autonomous vehicles, have become an innovative aspect of safety and luxury in modern vehicles [28]. State-of-the-art ADAS technologies mainly comprise cameras, light detection (lidar), radio detection (radar), ultrasonic sensors, and other advanced-sensing technologies. Moreover, the physiological sensing of drivers is implemented as an assistive technology for ADAS. EEG has been investigated for understanding driving behavior, drowsiness, and fatigue [26,29,30]. ECG-derived heart rate variability (HRV) was also used for driver drowsiness detection [31]. Multi-modal biosensors have also been utilized for understanding driver stress and driving style recognition [27,32]. Among EEG-based studies, neurological biomarkers have not yet been explored in response to driving-induced mental workloads.

Statistical significance and correlation analyses were not extensively explored for EEG spectral components in the previously reported driving simulator EEG studies. The identification of neurological biomarkers is essential to develop EEG-based ADAS systems. Numerous studies have presented machine-learning or deep-learning models without statistical interpretation of the role of the EEG features in the driving workload. It would be useful to have a detailed study combining a statistical analysis, hypothesis testing, correlation analysis, and machine-learning-based mental workload classification. In general, flat monitor or 180-degree virtual driving screens have been used in driver simulator studies, which lack a real-driving experience, compared to 360-degree screens. It would be an interesting to perform a study that reports driving EEG mental-workload data in a motion driving simulator equipped with a 360 degrees full-screen actual car cabin and that provides an near real-driving experience.

We hypothesized that the responses of the central nervous system affected by cognitive workload from driving would be immediately sensed by the EEG circuitry. Signal processing-based feature extraction, followed by statistical data analysis, is likely a reliable method to explore the physiological and functional outcomes of driving neurological workloads.

We aimed to investigate the driver’s EEG activity and identify the physiological biomarkers while driving in various road scenarios. We developed a neurological state prediction model to classify the neurological responses in different driving environments. The key contributions of this paper can be summarized as follows:

- We established an EEG-based driver-assistance system and driving advisor recommendation platform, integrating a wearable EEG sensor, data streaming to a cloud server, real-time signal processing, service dashboards for the drivers, and service managers for driving recommendations and health advisor support.
- We identified EEG biomarkers, including frequency spectral measures, while driving with route-induced cognitive demands, using statistical analysis and hypothesis tests.
- We developed machine-learning models to classify the neurological states affected by the cognitive demands in changing traffic environments.

We organized the remainder of this article into four sections. The proposed EEG-based driver-assistance system and driving advisor platform, followed by the datasets and the methodology used to validate the system’s predictive capability, are described in Section 2. After that, the results are reported in Section 3, followed by a discussion. Lastly, we state the conclusions in Section 5.

## 2. Materials and Methods

### 2.1. EEG-Based ADAS System

**Figure 1:**
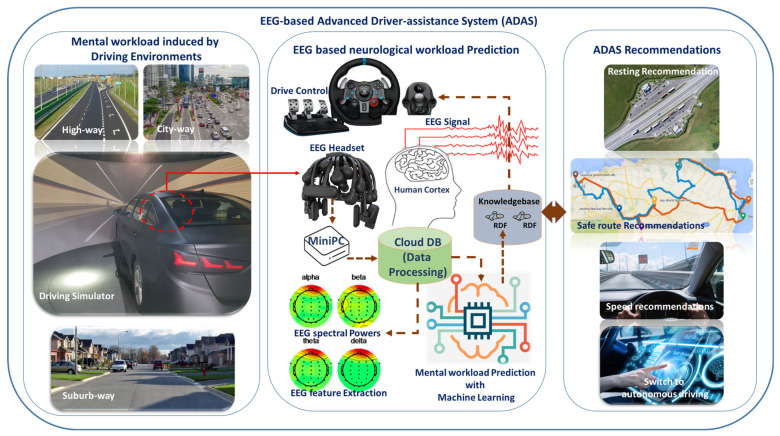
Overview of an EEG-based advanced driver-assistance system (ADAS). The EEG-based ADAS system consists of the wearable EEG headset, a car-embedded MiniPC for acquisition and transmission of data, Cloud DB for live data processing, and a relational DB (RDB) for providing a query service between the driving knowledgebase framework and front-end ADAS service applications. This ADAS system was developed to identify the changes in neurological states due to driving workloads and generate driving recommendations to assist the drivers.

**Figure 2:**
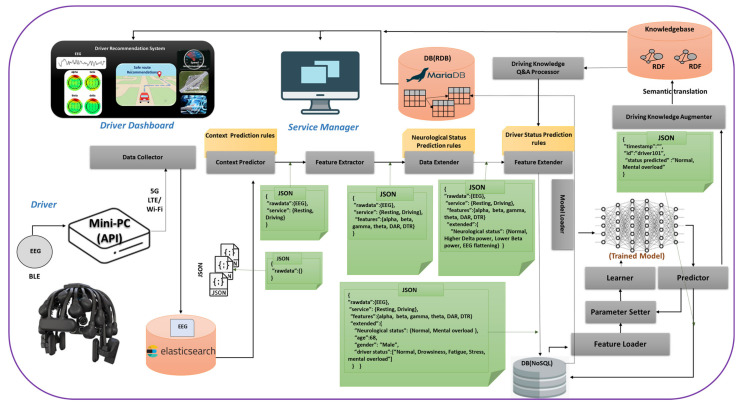
The dataflow of the EEG-based ADAS system. The system feeds the EEG data to a cloud server through a Wi-Fi or LTE network using ActiveMQ queue. In the cloud server, Elasticsearch indexes and stores the data; and Spark performs live data processing, such as context prediction, feature extraction, rule-based feature extension, and machine learning-based prediction. EEG features with disease prediction are fed to the machine learning model for training the model, to build a driver neurological state prediction engine. RDB stores the processed data and provides a query service for front-end service applications. Driving ontology will assist in understanding the correlation of physiology and mental workload with the driving conditions and driving information. ADAS can recommend safe driving suggestions through the service dashboard.

An EEG-based advanced driver-assistance system (ADAS) was proposed to predict the mental workload of drivers in varied driving scenarios. As demonstrated in Figure 1, this system consisted of a wireless EEG headset, a data transfer interface, a networking gateway, cloud storage and processing, and a physiological analytics service. The EEG headset generates neurological data as health level 7 (HL7 V2) messages, produced according to the protocol of the standards of HL7 International, and sent to the Elasticsearch database (DB) through a Wi-Fi or LTE network. Elasticsearch performs data indexing and stores the data in a No-SQL database. The feature extraction, feature selection, and machine learning algorithms were implemented in the Apache Spark platform for real-time processing. Wearable devices generate a vast amount of data and can be characterized in terms of ‘3 Vs’ (volume, variety, and velocity) for big data [33,34]. These data need to be processed using big-data-based processing for real-time healthcare services [6,7,35,36]. We utilized the Apache Hadoop platform for the online processing of wearable big health data. Relevant neurological features, such as spectral power measures, were extracted using feature extraction algorithms. In the next step, feature selection provided the key features of mental workload and reduced the computational time, by eliminating unnecessary features for training the machine learning models. The selected neurological features were fed to the ML model for training classification models. The driving knowledgebase showed the related mental workload, stress, and driving complications as assistance for safe driving, route recommendation, and switching to autonomous driving if available. All processed data could be visualized in dedicated monitors for drivers and ADAS service providers. The data flow of the EEG-based ADAS system is displayed in Figure 2.

### 2.2. Study Design

**Figure 3:**
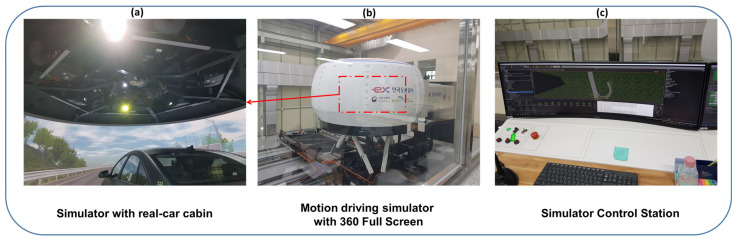
Motion driving simulator used in this study. (**a**) 360 full-screen real car cabin inside the driving simulator, (**b**) exterior view of driving simulator displaying the car cabin and the electronic XY rail system (20 × 9 m), which can achieve a high level 0.7G acceleration, (**c**) driving simulator control station.

The study was conducted according to a protocol approved by the Institutional Review Board of Korea Research Institute of Standards and Science, Daejeon, South Korea. A driving simulator, located in the Korea Expressway Corporation, Driving Simulator Center, Hwaseong, Gyeonggi-do, South Korea, was utilized in this study. The driving simulator and virtual reality were designed by Innosimulation Co., Ltd., Seoul, South Korea, and SCANeR studio ver. 1.8 was utilized for the acquisition of driving information. This simulator consists of a 360° Full-Screen Real Car Cabin, Electronic XY Rail System (20 × 9 m), which can achieve a level of acceleration up to 0.7 G, as shown in Figure 3. The simulator was operated in a room under a constant room temperature (25 °C) and humidity (40–50%). Before the start of the experiment, the experimental scenario was explained to the participants. Several practices were allowed for each participant to adjust to the simulator. Sufficient rest time was given between practicing and the real test. The driving scenario in the experiment consisted of several task elements, such as starting, stopping, and right and left turns. Every participant drove two types of driving routes, expressway and city-roadway routes. The EEG headset, electrooculogram (EOG), and electromyogram (EMG) electrodes were attached to the participants, followed by resting for 10 min. Measurement of EOG and EMG was performed only in the resting state for removing eye-blinking and muscular artifacts from the EEG signal. Data were recorded during pre-driving resting state for one minute, five minutes in the city-roadway state, and four minutes in the expressway driving state. A five-minute rest-time was provided between each segments of tasks.

### 2.3. Driving Scenarios and Mental Workload

Every participant drove two types of driving routes, expressway and city-roadway routes. In the expressway low-workload scenario, participants were required to drive on a straight and uniform road. There was no turning and no change of direction available. Adapting their speed relative to driving conditions was the main task in this scenario. Participants were instructed to maintain varying posted speed limits (from 60 km/h to 100 km/h). Participants were encouraged to be economical in their driving while driving along the route within the encountered speed limit. In the city-roadway high-workload scenario, participants were instructed to drive along a straight, flat, and non-priority road. Adapting their speed relative to city-way heavy traffic conditions was the main task in this scenario. The city-roadway consisted of an intersection, frequent lane-changes, and signals at the intersection. Participants were instructed to maintain a speed ranging from 40 km/h to 60 km/h, not to make left or right turns, and follow all traffic rules. Subjective measures of perceived mental workload were gathered from the participants after the driving tasks using a NASA task load index (NASA-TLX) questionnaire [37].

### 2.4. Participants in the Experiment

The volunteers who participated in this experiment were twenty-three healthy male drivers, having an average age of 65.2 ± 3.27 years and driving experience of 31.5 ± 10.75 years. Five participants’ data could not be processed due to excessive noise, and one participant failed to complete the test. The dataset utilized in this experiment was EEG data recorded from seventeen participants who successfully finished the driving tasks according to the experiment protocol. All the participants performed three simulated driving tasks consecutively, with simultaneous recording of physiological parameters. The participants had no clinical history of mental diseases or visual problem with driving a car in the driving simulator. The participants had no clinical history of any known neurological diseases. EEG data were processed at the Center for Medical Convergence Metrology, Korea Research Institute of Standards and Science, Daejeon, South Korea.

### 2.5. Data Acquisition

**Figure 4:**
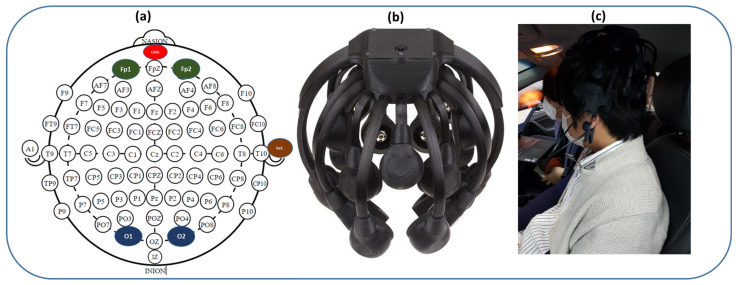
EEG headset and electrode position layout. (**a**) Four-channel EEG (Fp1, Fp2, O1, O2), reference (A2), and ground (Nz) electrodes position based on a standard 10–20 EEG system, (**b**) Cognionics Quick-20 EEG headset, (**c**) experimental scenario.

In this study, EEG was recorded as the representative signal of the neural system of the brain. Four-channel EEG data were acquired using a Cognionics Quick-20 EEG headset (Cognionics, Inc., San Diego, CA, USA), with Cognionics data acquisition 2.0 software at a sampling rate of 1000 Hz. The amplifier captured the signal via dry comb (no gel/skin preparation) electrodes attached to the scalp. Each dry electrode, coupled with a local active amplifier and Faraday cage, enables high-quality signal acquisition, despite a higher electrode impedance due to dry skin contact. In this study, EEG data were taken on the Fp1, Fp2, O1, and O2 positions, according to the international 10–20 EEG system, as shown in Figure 4. Fp1 and Fp2 are representative of the frontal lobe, and O1 and O2 are representative of the occipital lobe. The average EEG measurement of the frontal and occipital channels was considered the global channel, representing the entire cortex. In addition, two channels of EOG were recorded in the left and right eyes using a Biopac MP 160 System (Biopac Systems Inc., Goleta, CA, USA) and AcqKnowledge ver. 5.0 software (Biopac Systems Inc., Goleta, CA, USA) in the resting state to filter eye blinks, a single-channel chin EMG was recorded using a Myoresearch DTS System (Noraxon Inc., Scottsdale, AZ, USA), while MR3 Myomuscle software (Noraxon Inc., Scottsdale, AZ, USA) was used in the resting state to remove muscular artifacts. Participants were kept in the resting state for five minutes, performed driving for around eight minutes in the city-roadway state, and around six minutes in the expressway driving state. EEG data were recorded for one minute in the resting state, five minutes in the city-roadway state and, four minutes in the expressway driving state. The data from the first half minute were removed for each task to remove unstable signals due to switching operations.

### 2.6. Pre-Processing

60 Hz AC noise of the local electrical grid was filtered out of the EEG signal. Artifacts from the electrooculography (EOG) and electromyography (EMG) signals were filtered out of the EEG signal. EEG data were downsampled to 250 Hz to reduce computational time. Independent component analysis (ICA) was utilized to remove ocular and muscular artifacts in the EEG. We used the FastICA algorithms for denoising the EEG signal [38]. ICA utilized EOG and EMG recordings to isolate the EEG waveform from the eye-blink and muscular artifacts. Low-frequency motion artifact noise was caused by movements of the head and the movement of the sensor relative to the skin. A signal-to-noise ratio (SNR) was estimated for each signal by taking the power ratio of the movement-effected EEG signal and the undisturbed measurement [39]. The EEG waveform was filtered within the 0.5–44 Hz frequency range using a band-pass filter. AcqKnowledge ver. 5.0 software (Biopac Systems Inc., Goleta, CA, USA) was used for pre-processing and feature extraction of EEG data.

### 2.7. Feature Extraction

EEG can be described in terms of the frequency and power within specific frequency bands, i.e., delta (δ) band ranges in 0.5–4.0 Hz, theta (θ) band ranges in 4.0–8.0 Hz, alpha (α) wave runs on 8.0–13.0 Hz, the beta (β) band maintained in 13.0–30.0 Hz, and gamma (γ) wave runs on 30.0–44.0 Hz. Different EEG features were isolated from the EEG signals using FFT and other techniques to examine the power within the EEG signals. For each time epoch, the power spectrum was estimated from the power spectral density (PSD) using the Welch periodogram method. From this PSD, the mean-power, median frequency, mean frequency, spectral edge, and peak frequency features were extracted during each epoch. The epoch width was characterized as 10 s. Table 1 lists all the EEG features extracted in this study. This EEG dataset consists of 51 sets of resting state EEG features, 459 sets of city-roadway driving EEG features, and 306 sets of expressway driving EEG features in total; 3 sets of resting EEG features, 27 sets of city-roadway driving EEG features, and 18 sets of city-roadway driving EEG features on average for each subject.

#### 2.7.1. EEG Frequency-Domain Features

The EEG frequency analysis was carried out using fast Fourier transformation (FFT) using a Welch periodogram [40] performed on an artifact-free EEG signal with 10% hamming and extracted absolute power in the following spectral frequency bands: delta band, δ (0.5–4.0 Hz), theta band, θ (4.0–8.0 Hz), alpha, α (8.0–13.0 Hz), beta band, β (13.0–30 Hz), and Gamma band, γ (30.0–44 Hz). The mean power was defined as the average power of the power spectrum within the epoch. The median frequency was defined as the frequency at which 50% of the total power within the epoch was reached. The mean-frequency was defined as the frequency at which the average power within the epoch was reached. The spectral-edge was defined as the frequency below which 90% of the total power within the epoch was reached. The peak-frequency was defined as the frequency at which the maximum power occurred during the epoch. EEG relative power (RP) was calculated as the ratio of each band power to the total power of all bands, to normalize the amplitudes of individual EEG bands. All band powers were extracted with an epoch length of 10 s. The spectral power density of EEG time-series signal x(t) with frequency j, is defined as follows:

(1) $$E_{j}=\lim_{t\to \infty }\frac{1}{t}{\left|\hat{x_{t}}\left(j\right)\right|}^{2}$$

where $\hat{x_{t}}\left(j\right)$ is the fourier transform of x(t) at frequency j (in Hz) using Welch periodogram. EEG band relative power is defined as

(2) $$e_{j}=\frac{E_{\left(j_{1},j_{2}\right)}}{\sum_{j=0.5}^{44}E_{j}}$$

where E_j_ is the absolute spectral power density with frequency j (with j = 0.5, …, 44), and j_1_ and j_2_ are the low and high frequencies in Hz, respectively, and (j_1_, j_2_) is defined as δ (0.5, 4), θ (4, 8), α (8, 13), β (13, 30), and γ (30.0, 44). The changes of EEG power on driving cognitive states relative to the resting state help understand the characteristics of the neurological phase changes for different driving environments. At first, the average EEG power of each band was derived in the resting state. The average resting EEG band power, ${\bar{\mathrm{re}}}_{r}$ was calculated as follows,

(3) $${\bar{\mathrm{re}}}_{r}=\frac{\sum_{k=1}^{n}{\mathrm{re}}_{k}}{n}$$

where re_k_ is the relative power on each epoch. Then the change of EEG power during driving states (city-roadway and expressway driving tasks) relative to the resting state (baseline) $∆e_{m}$ was calculated as follows,

(4) $$∆e_{m}=\frac{\left(e-{\bar{\mathrm{re}}}_{r}\right)}{{\bar{\mathrm{re}}}_{r}}*100\%$$

where e is the EEG band relative power in different driving states (resting, city-roadway, and expressway driving), ${\bar{\mathrm{re}}}_{r\;}$ is the average resting EEG band power, and m is the level of task, such as resting, city-roadway and expressway driving.

EEG asymmetry is a connectivity measure that reflects the relative activity of spectral components between the left and right brain hemispheres and is determined as the relative difference between the power of the spectral waves of the right and left hemispheres and the total power of both hemispheres [41,42]. In this study, only frontal EEG asymmetry was measured, the and relative difference of the spectral features of frontal EEG electrodes (Fp1 and Fp2) was described as frontal EEG asymmetry. Fp1 is a representative electrode of the left hemisphere, and Fp2 is a representative electrode of the right hemisphere. EEG asymmetry is defined as

(5) $$A_{j}=\frac{\left|e_{j_{R}}-e_{j_{L}}\right|}{\left|e_{j_{R}}+e_{j_{L}}\right|}$$

where $e_{j_{R}}$ and $e_{j_{L}}$ are the spectral power of the right and left hemisphere EEG signals.

#### 2.7.2. DAR and DTR

The ratio of delta power and alpha band power was computed to measure the DAR (delta-alpha ratio). The ratio of delta power and theta band power was defined as the delta-theta ratio (DTR).

(6) $$\mathrm{DAR}=\frac{e_{j=\delta }}{e_{j=\alpha }}$$

(7) $$\mathrm{DTR}=\frac{e_{j=\delta }}{e_{j=\theta }}$$

where j is the spectral frequency range, delta (δ) ranges 0.5–4.0 Hz, theta (θ) ranges 4.0–8.0 Hz, and alpha (α) ranges 8.0–13.0 Hz; $e_{j=\delta }$ and $e_{j=\alpha }$ are the EEG band relative delta and alpha powers, respectively, in different driving states (resting, city-roadway and expressway driving.

### 2.8. Features Selection

Feature selection minimizes the data processing time and memory usage, allowing machine learning algorithms to handle only the significant features. Feature importance, ranging from zero to one, was calculated using F-statistics [43]. We performed a one-way ANOVA F-test for each continuous predictor and utilized the *p*-value based on F-statistics for feature selection to explore the most-contributing features. The features with constant and missing values were screened out in an earlier step. The feature importance was determined based on the effectiveness of each feature, predicting the target class independently. Features with a feature importance (1–*p*) greater than 95% were selected in this process, where *p* is the F-test outcome.

### 2.9. Classification Algorithms

Machine-learning algorithms were utilized for the classification of the neurological features during a resting state and driving along the city-roadway and expressway. The EEG feature data of the twelve participating drivers were labeled as training datasets, while EEG feature data of five drivers were kept as testing dataset. A k-nearest neighbors model (KNN), discriminant analysis model, support vector machine (SVM), C5.0, and QUEST model were implemented to discriminate neurological features of the resting state and driving states. SVM maps data onto a high-dimensional feature space, so that features can be categorized by generating a marginal line. The C5.0 model is a supervised data mining tool used to build decision trees using a divide-and-conquer method. As the resting-state dataset was smaller than the driving state datasets, we implemented a ‘class weighting’ technique [44], heavily weighting the resting-classes and under-weighting the majority classes to deal with the imbalance dataset.

#### 2.9.1. k-Nearest Neighbors Model (KNN)

The k-nearest neighbors model is a simple algorithm that classifies cases based on similarity measures (e.g., distance functions) to other cases. KNN has been used in statistical estimation and pattern recognition as a non-parametric technique. k is described as the number of nearest neighbors. In this study, we specified that k = 3.

#### 2.9.2. Discriminant Analysis Model

The discriminant analysis model forms a predictive model for group memberships. The model generates a discriminant function based on linear combinations of the predictor features that provide the best classification between the target classes [45].

#### 2.9.3. SVM Model

The support vector machine (SVM) is a widely used classification model that maps data by forming a higher dimensional hyperplane, so that features can be classified by creating a margin line using a popular kernel method, Gaussian kernel; an example of a radial basis function (RBF) kernel. We trained the SVM model and performed k-fold cross-validation (k = 10). SVM is most appropriate for use with wide-ranging datasets with lots of input fields [46]. Gaussian kernel, is a widely used kernel function in various kernelized learning algorithms, such as SVM. This RBF kernel depends only on the distance between the feature vectors, instead of their position. The Gaussian kernel function is defined in Equation (8).

(8) $$\mathrm{Gaussian}\;\mathrm{Kernel},\;K\;\left(X_{1}-X_{2}\right)=\mathrm{exponent}\left(-{\gamma X}_{1}-X_{2}\right)$$

where ‖X_1_ − X_2_‖ is the Euclidean distance between two EEG feature vectors, X_1_ & X_2_; γ (gamma) is the inverse of the standard deviation of the RBF kernel (Gaussian function).

#### 2.9.4. C5.0 Model

The C5.0 model is a supervised data mining algorithm used to build decision trees or rule sets from data [47]. This model splits the data based on the field that provides the highest gain ratio. The model builds the decision tree, followed by a pruning procedure to minimize the tree’s estimation error rate. This model does not require a long training time for prediction and is robust for missing data and many input fields.

#### 2.9.5. Quick, Unbiased, and Efficient Statistical Tree (QUEST) Model

QUEST is a binary-split decision tree algorithm with univariate or linear combination splits and used for classification problems. The attribute selection of the QUEST model has negligible bias [48]. This tree growing process includes the selection of a split predictor, selection of a split point for the selected predictor, and stopping. In this algorithm, only univariate splits are considered.

### 2.10. Data Analysis

We compared the demographic data of the participants using descriptive statistics. The resting EEG, recorded before the driving tasks, was considered the baseline for this study. The EEG spectra features and relative changes of features in the driving tasks relative to the resting state are presented in a bar chart with an error bar. Data in the bar chart represent the mean value of each data with a corresponding 95% confidence interval (CI). A paired-samples *t*-test was used as a comparative measure of the means of data between the resting state and the driving state. A *p* value of less than 0.05 was considered statistically significant. Statistical analyses were performed using SPSS 24 software (IBM, Armonk, NY, USA). We utilized state-of-art machine learning algorithms to classify the resting state and other driving states. We partitioned the EEG feature datasets into two categories: the training dataset, and the testing dataset. Using the training dataset, we trained the machine learning algorithms to construct classification models, which were later used for prediction using the driving EEG testing datasets. We performed non-exhaustive k-fold (k = 10) cross-validation using the training dataset to get rid of overfitting. We used IBM SPSS Modeler 18 software (IBM, Armonk, New York, NY, USA) and TensorFlow [49] for machine learning analyses.

## 3. Results

### 3.1. Statistical Analysis

#### 3.1.1. Association of Driving Environments with EEG Features

**Figure 5:**
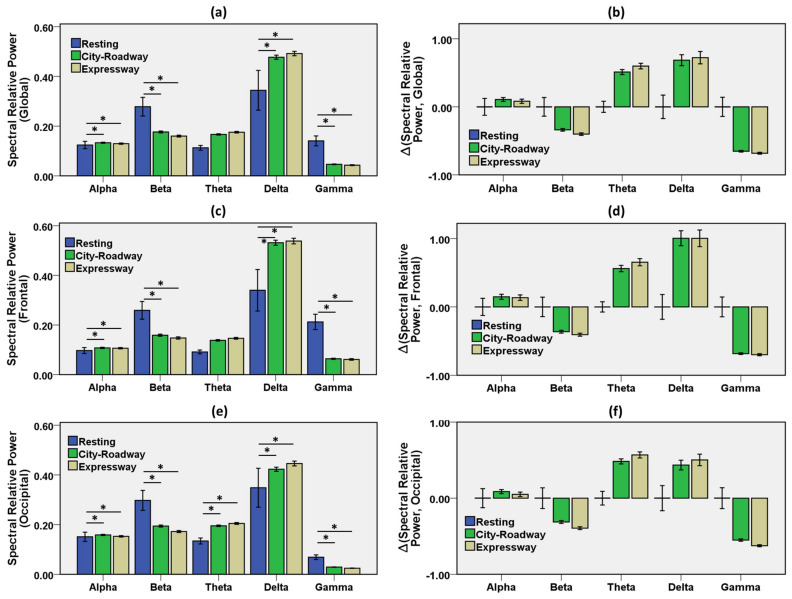
Results from EEG spectral power features during resting state and driving along the city-roadway and expressway. Global indicates the average measures of features of the frontal and occipital lobes. Bar describes the relative difference from baseline and error bar as the 96% CI, * indicates *p* < 0.05. (**a**) Global spectral features for resting and all driving states (*p* < 0.05). (**b**) Changes of the global spectral features in every driving scenario relative to resting state (*p* < 0.05). (**c**) Frontal spectral features for resting and all driving states (*p* < 0.05). (**d**) Changes of the frontal spectral features in each driving scenario relative to the resting state (*p* < 0.05). (**e**) Occipital spectral features for resting and the driving scenarios (*p* < 0.05). (**f**) Changes of the occipital spectral features in each driving scenario relative to the resting state (*p* < 0.05). ∆ Indicates the change relative to the baseline (the resting–state).

According to the NASA task load index (NASA-TLX) questionnaires, driving along the city-roadway demanded a higher mental workload relative to the expressway driving. EEG spectral power features were explored during a resting state and driving along the city-roadway and expressway (Table 2). The mental workload varied during driving with those scenarios. Figure 5 shows bar charts, with error bars with a 95% confidence interval (C.I.), of EEG features of the frequency bands during the resting, city-roadway, and expressway driving. Figure S1 demonstrates the frontal EEG asymmetry of features during the resting state and driving states. Global indicates the average measures of the features of the frontal and occipital lobes. Global alpha was lowest in the resting state. Global alpha increased 7.3% in the city-roadway driving and 4.8% in the expressway driving over the resting state. Frontal lobe alpha was lowest in the resting state. In addition, frontal alpha increased 11.3% in the city-roadway driving and 9.3% in the expressway scenario, relative to the resting state. Moreover, occipital alpha increased 5.3% in the city-roadway driving and 1.3% in the expressway over the resting state. Alpha power was a significant feature to distinguish the resting state and each driving scenario.

Global beta decreased −36.7% in the city-roadway driving and −42.4% in the expressway scenarios compared to the resting state. Moreover, frontal beta decreased −38.6% in the city-roadway driving and −42.9% in the expressway scenario relative to the resting state. Moreover, occipital beta decreased −34.7% in the city-roadway driving and −42.1% in the expressway compared to the resting state. Beta power was a significant feature for classifying the resting state and all driving states.

Global theta increased 47.8% in the city-roadway driving and 54.9% in the expressway scenario over the resting state. While, frontal theta increased 51.6% in the city-roadway driving and 60.4% in the expressway scenario relative to the resting state. Furthermore, occipital theta increased 45.5% in the city-roadway driving and 53.0% in the expressway compared to the resting state. Theta power showed significant differences in the occipital lobe and no significant differences in the frontal lobe, for distinguishing the resting state and driving states. As shown in Figure S1c, significant frontal theta asymmetry was observed between the resting state and city-roadway state; and between the city-roadway state and expressway state.

Global delta increased 38.7% in the city-roadway driving and 43.0% in the expressway scenario relative to the resting state. Moreover, the frontal delta increased 56.5% in the city-roadway driving and 58.2% in the expressway scenario relative to the resting state. Furthermore, the occipital delta increased 21.3% in the city-roadway driving and 28.2% in the expressway over the resting state. Delta power was a significant biomarker for classifying the resting state and driving states. As shown in Figure S1d, significant frontal delta asymmetry was observed between the resting state and city-roadway state.

Global gamma decreased −66.7% in the city-roadway driving and −69.5% in the expressway scenarios compared to the resting state. In addition, frontal gamma decreased −69.8% in the city-roadway driving and −71.2% in the expressway scenario relative to the resting state. Moreover, occipital gamma decreased −58.0% in the city-roadway driving and −63.8% in the expressway relative to the resting state. Gamma power was a significant parameter for distinguishing the resting state and driving states.

#### 3.1.2. Changes of DAR and DTR in the Driving States

**Figure 6:**
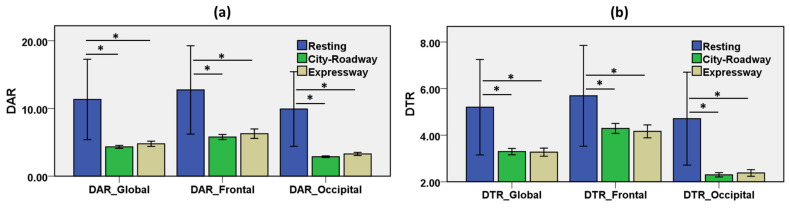
Results from DAR and DTR during resting state and driving along the city-roadway and expressway. Global indicates the average measures of features of the frontal and occipital lobes. Bar describes the relative difference from the baseline and error bar as the 96% CI, * indicates *p* < 0.05. (**a**) Global, frontal, and occipital DAR for resting and all driving states (*p* < 0.05). (**b**) Global, frontal, and occipital DTR for resting and all driving states (*p* < 0.05). ∆ Indicates the change relative to the baseline (the resting state).

Delta power ratios, such as DAR and DTR, were explored during the resting state and driving along the city-roadway and expressway (Table 3). The driving-induced mental workload varied with the scenarios. Figure 6 shows the bar charts with error bars with a 95% confidence interval of DAR and DTR during the resting, city-roadway, and expressway driving. DAR was dominant in the resting state. Global DAR decreased −61.8% in the city-roadway driving and −57.8% in the expressway scenario compared to the resting state. Moreover, frontal DAR decreased −54.6% in the city-roadway driving and −50.8% in the expressway scenario relative to the resting state. Furthermore, occipital DAR decreased −71.1% in the city-roadway driving and −66.9% in the expressway compared to the resting state. DAR was a significant feature for classifying the resting state and driving states.

DTR was dominant in the resting state. Global DTR decreased −36.6% in the city-roadway driving and −37.1% in the expressway scenario compared to the resting state. Furthermore, frontal DTR decreased −24.6% in the city-roadway driving and −26.8% in the expressway scenario relative to the resting state. Moreover, occipital DTR decreased −51.2% in the city-roadway driving and −49.5% in the expressway below the resting state. DTR was a significant biomarker for distinguishing the resting state and driving states.

#### 3.1.3. Correlation of Delta and Theta with Driving Workload

**Figure 7:**
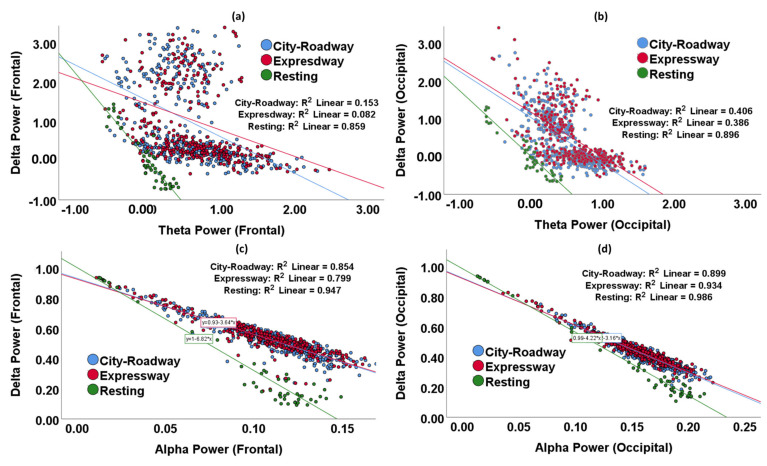
Scatterplot and regression line of delta and theta, and delta and alpha measures to understand the correlation of delta, alpha, and theta power while driving with various driving workloads (resting, the city-roadway, and expressway driving tasks). (**a**) Scatterplot and regression line of delta and theta features for the frontal lobe, (**b**) scatterplot and regression line of delta and theta features for the occipital lobe, (**c**) scatterplot and regression line of delta and alpha features for the frontal lobe, (**d**) scatterplot and regression line of delta and alpha features for the occipital lobe. R^2^ denotes the coefficient of determination, the slope of the regression line, r denotes the correlation coefficient.

As shown in Figure 7, a scatterplot and regression line of the delta and theta, and delta and alpha, features of the frontal and occipital lobes were explored to understand the correlation of delta power with the theta and alpha powers during varied driving workloads (resting, the city-roadway and expressway driving tasks).

Frontal delta and theta had strong negative correlations in the resting state (correlation coefficient, r = −2.24), in the city-roadway driving state (r = −0.92), and in the expressway driving state (r = −0.66). On the other hand, occipital delta and theta also showed strong negative correlations in the resting state (correlation coefficient, r = −1.74), in the city-roadway driving state (r = −1.22), and the expressway driving state (r = −1.18).

A total 86% of the resting frontal delta and theta data variations were determined by this regression line (coefficient of determination, R^2^ = 0.859, *p* < 0.001). The plotted regression lines described 8% of the city-roadway driving state data (R^2^ = 0.082, *p* = 0.15) and 15.3% of the expressway driving state data (R^2^ = 0.153, *p* < 0.001). On the other hand, 90% of the resting occipital delta and theta data variation was determined by this regression line (coefficient of determination, R^2^ = 0.896, *p* < 0.001). The plotted regression lines describe 41% of the city-roadway driving state data (R^2^ = 0.406, *p* < 0.001) and 39% of the expressway driving state data (R^2^ = 0.386, *p* < 0.001). Therefore, it was observed that the regression line of delta power and theta features was a better fit for the occipital lobe EEG in a resting state and all driving states.

Frontal delta and alpha had strong negative correlations in the resting state (correlation coefficient, r = −6.82), in the city-roadway driving state (r = −3.64), and in the expressway driving state (r = −3.68). On the other hand, occipital delta and alpha also showed strong negative correlations in the resting state (correlation coefficient, r = −4.22), in the city-roadway driving state (r = −3.16), and the expressway driving state (r = −3.12).

95% of the resting frontal delta and alpha data variations were determined by this regression line (coefficient of determination, R^2^ = 0.947, *p* < 0.001). The plotted regression lines describe 85% of the city-roadway driving state data (R^2^ = 0.854, *p* < 0.001) and 80% of the expressway driving state data (R^2^ = 0.799, *p* < 0.001). On the other hand, 99% of the resting occipital delta and alpha data variations were determined by this regression line (coefficient of determination, R^2^ = 0.986, *p* < 0.001). The plotted regression lines describe 90% of the city-roadway driving state data (R^2^ = 0.899, *p* < 0.001) and 93% of the expressway driving state data (R^2^ = 0.934, *p* < 0.001). Therefore, it was observed that the regression line of delta power and alpha features was also a better fit for the occipital lobe EEG in a resting state and all driving states.

### 3.2. Machine Learning Analysis

Machine-learning approaches were explored to predict the neurological states of drivers during the resting state and the city-roadway and the expressway driving states. Machine learning analysis is composed of feature selection, cross-validated model training, and model testing. In the feature selection process, the feature importance of driving EEG features was evaluated using F-statistics. EEG features having a feature importance with a *p*-value greater than 0.95 were selected for further classification study. A total of 112 features were chosen out of 149 initial extracted brainwave features, based on feature importance (*p* > 0.95). To assess the classification performance, a ROC (receiver operating characteristic) curve is one of the most effective tools. AUC (area under the curve) is defined as the area under the ROC curve, ranging from 0 to 1.0. The higher the AUC, the better the performance of the model. In addition, the Gini coefficient is an alternative model performance measure and is defined as two times (AUC-1), ranging between 0 and 1. The confusion matrix or error matrix provides a clear demonstration of the prediction results for all target classes. From the confusion matrix, several other performance parameters, such as accuracy, AUC, and Gini coefficient were calculated. The accuracy was calculated as the ratio of correct predictions to the total observations and considered the most intuitive performance measure to identify the best model. ROC curves display the sensitivity and specificity scores for each predictive model. The performance evaluation parameters were computed using the following standard formulas:

$$\mathrm{Accuracy}=\frac{\mathrm{TN}+\mathrm{TP}}{\mathrm{TN}+\mathrm{TP}+\mathrm{FN}+\mathrm{FP}}$$

where TP stands for the true positive, TN means the true negative, FP stands for the false positive, and FN means the false negative.

#### 3.2.1. Multi-Class Classification of Resting State and Driving States

We utilized machine learning algorithms for multi-class classification of the resting state and the driving states (resting, the city-roadway, and expressway driving tasks). The confusion matrices of five machine-learning models (KNN Model, C5.0, and SVM, discriminant analysis, QUEST models) are given in Table 4 as the outcomes of prediction for the resting state and driving states.

The KNN model showed a 76.73% accuracy using the training dataset and 64.23% accuracy using the testing dataset for multi-class classification of the resting and other driving states. Resting states were most accurately classified, with the accuracy for training (93.94%) and for testing (88.89%). Moreover, city-roadway driving states were classified with an accuracy for training (79.32%) and for testing (63.31%). Furthermore, expressway driving states were classified with accuracy for training (72.08%) and for testing (59.55%).

The discriminant analysis model showed a 72.01% accuracy using the training dataset and 66.26% accuracy using the testing dataset for multi-class classification of the resting and other driving states. Resting states were most accurately classified, with accuracy for training of (93.94%) and for testing (88.89%). Moreover, city-roadway driving states were classified with an accuracy for training of (72.54%) and for testing (62.59%). Furthermore, expressway driving states were classified with an accuracy for training (68.89%) and for testing (66.29%). Frontal delta and occipital beta features showed a 0.17 and 0.12 predictor importance, respectively, using this model.

The SVM model showed a 78.92% accuracy using the training dataset and 68.70% accuracy using the testing dataset for multi-class classification of the resting and other driving states. Resting states were most accurately classified with accuracy for training (100%) and for testing (94.44%). Moreover, City-Roadway driving states were classified with an accuracy for training (82.03%) and for testing (69.06%). Furthermore, expressway driving states were classified with an accuracy for training (72.83%) and for testing (62.92%).

The C5.0 model showed a 96.46% accuracy using the training dataset and 64.63% accuracy using the testing dataset for multi-class classification of the resting and other driving states. Resting states were most accurately classified, with an accuracy for training (100%) and for testing (88.89%). Moreover, city-roadway driving states were classified with an accuracy for training (96.61%) and for testing (58.99%). Furthermore, expressway driving states were classified with an accuracy for training (95.85%) and for testing (68.54%). Global gamma and occipital alpha features had a 0.27 and 0.10 predictor importance, respectively, using this model.

The QUEST model showed a 67.45% accuracy using the training dataset and 64.63% accuracy using the testing dataset for multi-class classification of the resting and other driving states. Resting states were most accurately classified, with an accuracy for training (100%) and for testing (100%). Moreover, city-roadway driving states were classified with an accuracy for training (73.56%) and for testing (63.31%). Furthermore, expressway driving states were classified with an accuracy for training (56.98%) and for testing (59.55%). Frontal gamma and frontal alpha features showed a 0.30 and 0.29 predictor importance, respectively, using this model.

#### 3.2.2. Binary Classification of Resting State and Driving States

**Figure 8:**
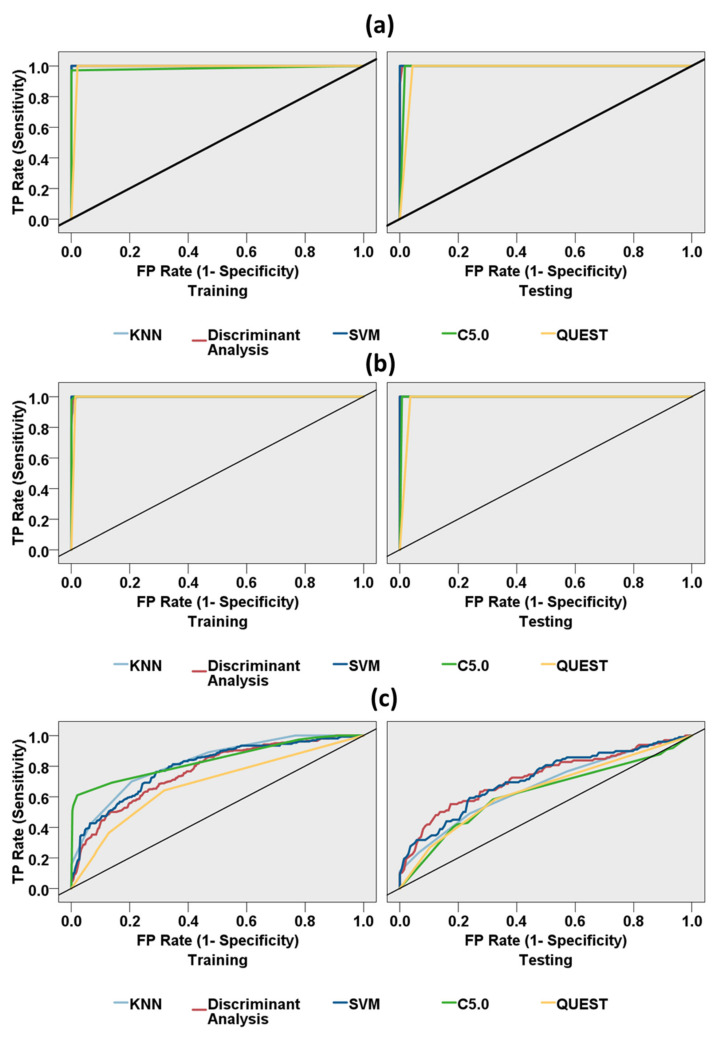
Receiver operating characteristic (ROC) curves for five different machine-learning models (k-nearest neighbors model, discriminant analysis model, support vector machine, C5.0, QUEST model). (**a**) ROC curve for binary classification of the resting and expressway datasets, (**b**) ROC curve for binary classification of the resting and city-roadway datasets, (**c**) ROC curve for binary classification of the city-roadway and expressway datasets. The area under the ROC curve (AUC) is an indicator of prediction accuracy. The diagonal black line is the reference line.

As displayed in Table 5, we utilized machine learning algorithms for binary classification of the resting state and the driving states (resting, the city-roadway, and expressway driving tasks). In addition, the feature predictor importance, ranging from zero to one, was evaluated to explore the contribution of features for classification using each model. Moreover, the ROC performance curves of five machine-learning models are demonstrated in Figure 8 for the one-to-one prediction performance for the resting state and other driving states.

As demonstrated in Table 5(a), the SVM model classified the resting and the expressway driving states with the highest accuracy (99.51%), a perfect AUC (1.0), and a perfect Gini coefficient (1.0). The KNN Model, C5.0, and discriminant analysis algorithms also showed near perfect accuracies in classifying the resting and expressway driving states. The ROC performance curves of all models for predicting the resting and expressway driving states are exhibited in Figure 8a. Frontal delta and occipital beta features showed a 0.19 and 0.14 predictor importance, respectively, using the discriminant analysis model. Occipital alpha and frontal delta features showed a 0.77 and 0.23 predictor importance, respectively, using the C5.0 model. The frontal alpha mean-frequency feature showed a 0.81 predictor importance using the QUEST model.

As displayed in Table 5(b), the SVM model classified the resting and the city-roadway driving states with the highest accuracy (99.79%), a perfect AUC (1.0), and a perfect Gini coefficient (1.0). The C5.0 and discriminant analysis algorithms also showed near-perfect accuracies, AUC, and Gini measures in classifying the resting and expressway driving states. The ROC performance curves of all models for predicting the resting and expressway driving states are exhibited in Figure 8b. The frontal delta and frontal theta features showed a 0.13 and 0.08 predictor importance, respectively, using the discriminant analysis model. Occipital beta and occipital gamma median-frequency features showed a 0.63 and 0.37 predictor importance, respectively, using the C5.0 model. The frontal beta mean-frequency feature showed a 0.83 predictor importance using the QUEST model.

As showed in Table 5(c), the C5.0 model classified the city-roadway driving and expressway driving states with the highest accuracy (75.38%), AUC (0.771), and Gini coefficient (0.541). The KNN, SVM, discriminant analysis, and QUEST algorithms also showed an accuracy of 71.95%, 69.54%, 68.27%, and 65.48%, respectively, in classifying the city-roadway driving and expressway driving states. The ROC performance curves of all models for predicting the city-roadway driving and expressway driving states are displayed in Figure 8c. The change of occipital beta relative to the resting state feature showed a 0.19 predictor importance using the discriminant analysis model. Frontal gamma mean-frequency and frontal theta median-frequency features showed a 0.15 and 0.11 predictor importance, respectively, using the C5.0 model. Frontal gamma mean-frequency and frontal gamma peak frequency features showed a 0.15 and 0.14 predictor importance, respectively, using the QUEST model. We performed repeated k-fold cross-validation with three additional repeats. We obtained similar accuracy results for those repeated k-fold cross-validations. The repeated k-fold cross-validation results are presented in the Supplementary section (Table S1a–c).

## 4. Discussion

In our study, we characterized the neurological changes of drivers due to various mental workloads while driving in different driving scenarios in a simulator. The extent of changes depended on the type of road and the speed and signal regulations, the behavior of surrounding vehicles, and traffic conditions overall. We evaluated the neurological biomarkers resulting from a resting state and driving during two driving tasks. As the resting-state possessed no mental workload due to driving, the resting state was characterized as the baseline for the study. A moderate mental workload was observed in the expressway driving, and a high mental workload was observed in the city-roadway driving.

Driving is a complex mental task, which involves challenges for the detection, perception, and processing of the information, as well as the skill demands, and constantly-changing visual and attentional activities. The central nervous system handles this mental workload and processes this complex activity in various cortical regions, based on the type of cognitive demand. As visual tasks are handled by the occipital cortex and the decision-making tasks are primarily processed in the frontal cortical region, the cortical region generating neurological responses varies based on the type and amount of driving mental workload.

EEG spectral power features were investigated during resting state and driving along a city-roadway and expressway in a simulated environment. The neurological outcomes of the primary driving task were affected by the drivers’ increased mental workload, imposed by secondary cognitive workloads. Identifying and quantifying the mental workload may be effective for ADAS, such as driver risk reduction, automated routes, resting-area recommendations, driving advisors, and so on.

Alpha frequencies are supposed to originate from cortical layers and appear during the eye-closed relax state [50]. The alpha power was lower in the resting state compared to the driving state. Beta activity increases with the degree of attention in tasks, and the city-roadway driving demanded greater attention relative to the expressway task. There was a slight decrease in beta activity compared to the resting pre-driving state. Among the driving modes, the beta activity showed no significant change in city-roadways driving relative to expressway driving. A decrease in beta activity could be seen when attentional activity was reduced while driving. EEG theta oscillation is involved in higher brain functions, including the working memory, executive control, and focused attention [51]. In this study, occipital theta showed significant increases in the driving states compared with the resting state. Previous studies also showed that the theta band changes with changes of various driving tasks, indicating an increase in mental workload [50,52,53,54]. Weaker delta activity was observed in the resting state relative to driving tasks and an increase of delta activity was seen in both cortical lobes in driving states compared with resting states. The slow-wave delta activity is considered the most reliable measure of mental workload relative to faster wave activity [55] and delta activity increased in the frontal area as driving progressed [17,56].

In the resting state, delta and theta had stronger negative correlations compared with the driving states in both frontal and occipital lobes. Similarly, delta and alpha had stronger negative correlations in the resting state compared with the driving states in both frontal and occipital lobes. Therefore, alpha, delta, and theta can be considered biomarkers of driving-induced mental workload among EEG spectral features. DAR and DTR were reported as a significant feature for assessing neural-impairment due to stroke [15], mental workload [17], and cognitive stress [57]. DAR and DTR showed significant differences between the resting and driving states in this study in the frontal and occipital regions. This outcome is supported by Xia et al. identifying a significant difference in the relative power ratios (DAR, DTR) between the stress and control conditions in the frontal and lateral lobes [57].

The asymmetric nature of the neurological response was observed in driving tasks in driving environments with varied mental demands. Previous studies reported that an increase in frontal EEG spectral asymmetry was associated with brain engagement in cognitive processes such as mental workloads, emotion, depression, and stress [41,42,52,58,59,60]. According to this study, a significant frontal theta asymmetry was observed between the resting state and city-roadway state; and between the city-roadway state and expressway state. This outcome is supported by previous findings, which identified an increase of theta power in the right hemisphere of the frontal lobe with an increase of mental workload [52,57]. Driving in the city-roadway entails repeated visual and decision-making tasks due to complex traffic systems and the behavior of surrounding cars. On the other hand, driving in the expressway mainly demands visual driving attentional tasks, and the frequency of decision-making tasks is limited. These asymmetric characteristics were reflected in the EEG spectral components in the frontal lobes. A higher theta asymmetry, observed in the frontal lobe in the city-roadway compared with resting and the expressway driving, is a neurological measure of mental workload while driving.

In the machine-learning study, the binary classification models showed near-perfect accuracy between the resting state and driving state, as expected. The EEG features varied greatly with the change of mental workload and cognitive task, and showed a clear discriminative pattern between the resting and driving neurological states. However, the machine-learning classifiers showed a moderate accuracy between the two driving states. A similar classifier performance was observed in the multi-class classification between the resting state, city-roadways state, and expressway state. Although the mental workload varied between the city-roadways and the expressways and although driving in city-roadways demands a higher cognitive workload than expressway driving, neurological variations still cannot be perfectly distinguished due to the complicated nature of cognitive demands [29].

Physiological signals were utilized for understanding fatigue, drowsiness, and stress in a driving context [54]. EOG and EEG were investigated to classify driving fatigue based on entropy analysis [27]. ECG-derived heart rate variability (HRV) was used for driver drowsiness detection [31]. EEG has been investigated for understanding driving behavior, drowsiness, and fatigue [26,29,30]. Table 6 demonstrates a comparative study of methodologies and results between the current work and EEG-related studies in different domains.

To our knowledge, this article is the first study that reports driving EEG data in a motion driving simulator equipped with a 360 degree full-screen real car cabin and electronic XY rail system, which can achieve a 0.7 G acceleration and provides an almost real driving experience. Most driving EEG studies present machine-learning or deep-learning models without interpretation of the role of EEG features in driving workload [54]. This study extensively explored EEG spectral components and their role in the neurological response in driving. We provided detailed statistical analysis along with hypothesis testing and evaluated EEG biomarkers contributing to mental workload during driving. We also explored the correlations of spectral features with different modes of driving.

EEG spectral measures are the indicators of mental and cognitive workload derived from tasks [17]. Driver mental workload should be quantified to maintain decent driving performance for the driver and road safety [53,54]. Driving-induced neurological parameters can be considered biomarkers for driver mental workload and guide the ADAS for safe driving recommendations, such as safe route selection, speed recommendation, resting-area suggestions, and other safe-driving recommendations. Although we analyzed EEG data based on the frontal lobe and occipital lobe to simplify neurological changes in EEG due to various driving conditions, we did not study each cortical lobe, such as the central lobe, parietal lobe, and temporal lobe, separately in order to understand the brain map. In addition, the dataset utilized in this study was relatively small, consisting of EEG data from seventeen drivers in different driving environments. In the future, we plan to extend this study with a larger number of participants to generalize this approach. Moreover, the resting state dataset was smaller compared with the driving state dataset. Although we implemented the class weighting technique to deal with the imbalanced dataset, we will record resting state data longer, to generate a balanced dataset. Moreover, this study was performed in a dynamic motion driving simulator. However, simulator driving may vary compared with a real driving situation. In the future, we will explore other physiological features, such as cardiac features, in various driving environments and traffic conditions, and determine effective physiological measures capable of assisting the ADAS system.

## 5. Conclusions

The prediction of driving-induced neurological workload is considered an assistive technology for advanced driver-assistance systems, for the driver and road safety. The neurological biomarkers of driving mental workload were quantified using a portable EEG headset in a motion driving simulator. In the driving state, the rise of theta and delta waves and a drop in the beta and gamma bands were observed relative to the resting state. Among the EEG spectral features, alpha, delta, theta, and frontal theta asymmetry can be considered biomarkers of driving-induced mental workload. Delta-derived measures, such as DAR and DTR, showed a strong correlation with resting state and different driving states. The binary machine-learning classification models showed near-perfect accuracy (around 98.2 to 99.6 percent) between the resting state and driving state. Moderate classification performances (around 65 to 75 percent accuracy) were observed in the multi-class classification between the resting state, city-roadways state, and expressway state driving. This EEG-based driving workload prediction technique is a promising candidate for ADAS and future neuroscience research in autonomous vehicle technology.

## Figures and Tables

**Table 1:** Features extracted from the EEG signal. The frontal channel is averaged over Fp1 and Fp2, and the occipital channel is averaged over O1 and O2 electrodes. The global channel is averaged over the Fp1, Fp2, O1, and O2 electrodes.

EEG Channel	EEG Spectral Waves	EEG Feature	Number of Features
Fp1, Fp2, O1, and O2	δ, θ, α, β, γ	Mean Power	20
Fp1, Fp2, O1, and O2	δ, θ, α, β, γ	Median Frequency	20
Fp1, Fp2, O1, and O2	δ, θ, α, β, γ	Mean Frequency	20
Fp1, Fp2, O1, and O2	δ, θ, α, β, γ	Spectral Edge	20
Fp1, Fp2, O1, and O2	δ, θ, α, β, γ	Peak Frequency	20
Global	δ, θ, α, β, γ	Mean Power	5
Frontal and Occipital	δ, θ, α, β, γ	Mean Power	10
Frontal and Occipital	δ, θ, α, β, γ	Change of Mean Power relative to Resting state	10
Fp1, Fp2, O1, and O2	DAR (δ/α), DTR (δ/θ)	Mean Power	8
Fp1, Fp2, O1, and O2	DAR (δ/α), DTR (δ/θ)	Change of Mean Power relative to Resting state	8
Fp1, Fp2, O1, and O2	-	Total Mean Power	4
Fp1, Fp2, O1, and O2	-	Change of Mean Power relative to Resting state	4

**Table 2:** Results of the statistical analysis of the Global EEG spectral power features during resting state (R) and driving along the city-roadways (C) and expressway (E). Global indicates averaging the frontal lobe and occipital lobe electrodes. Global EEG features are the average of the frontal and the occipital lobes. The resting state is considered the baseline. * indicates *p* < 0.05.

EEG Feature (Global)	Driving States	Mean Value	Standard Deviation	Relative Difference of C, E from Baseline (R), (C-R)/R or (E-R)/R	*t*-Test Significance, *p*-Value
Alpha (Relative Power)	Resting (R)	0.124	0.053	-	-
	City-Roadway (C)	0.133	0.024	0.073	0.04 *
	Expressway (E)	0.130	0.024	0.048	0.03 *
Beta (Relative Power)	Resting (R)	0.278	0.133	-	-
	City-Roadway (C)	0.176	0.040	−0.367	0.0001 *
	Expressway (E)	0.160	0.036	−0.424	0.0001 *
Theta (Relative Power)	Resting (R)	0.113	0.034	-	-
	City-Roadway (C)	0.167	0.029	0.478	0.06 *
	Expressway (E)	0.175	0.031	0.549	0.07 *
Delta (Relative Power)	Resting (R)	0.344	0.284	-	-
	City-Roadway (C)	0.477	0.088	0.387	0.0001 *
	Expressway (E)	0.492	0.085	0.430	0.0001 *
Gamma (Relative Power)	Resting (R)	0.141	0.071	-	-
	City-Roadway (C)	0.047	0.016	−0.667	0.0001 *
	Expressway (E)	0.043	0.017	−0.695	0.0001 *

**Table 3:** Results of the statistical analysis of the Global DAR and DTR during resting state (R) and driving along the city-roadway (C) and expressway (E). Global indicates averaging the frontal lobe and occipital lobe electrodes. The resting state is considered the baseline. * indicates *p* < 0.05.

EEG Feature (Global)	Driving States	Mean Value	Standard Deviation	Relative Difference of C, E from Baseline (R), (C−R)/R or (E-R)/R	*t*-Test, Significance, *p*-Value
DAR	Resting (R)	11.330	21.123	-	-
	City-Roadway (C)	4.324	2.345	−0.618	0.0001 *
	Expressway (E)	4.776	3.692	−0.578	0.0001 *
DTR	Resting (R)	5.200	7.281	-	-
	City-Roadway (C)	3.295	1.451	−0.366	0.0001 *
	Expressway (E)	3.271	1.670	−0.371	0.0001 *

**Table 4:** Confusion matrices of KNN Model, Discriminant Analysis, SVM, C5.0, and QUEST Model for classification of EEG features of the resting, the city-roadway driving, and expressway driving states.

		Prediction							
**Actual**	**KNN Model**	**Training (Accuracy = 76.73%)**				**Testing (Accuracy = 64.23%)**			
		**City-Roadway**	**Expressway**	**Resting**	**Accuracy**	**City-Roadway**	**Expressway**	**Resting**	**Accuracy**
	**City-Roadway**	234	59	2	79.32%	88	50	1	63.31%
	**Expressway**	74	191	0	72.08%	36	53	0	59.55%
	**Resting**	2	0	31	93.94%	2	0	16	88.89%
**Actual**	**Discriminant Analysis Model**	**Training (Accuracy = 72.01%)**				**Testing (Accuracy = 66.26%)**			
		**City-Roadway**	**Expressway**	**Resting**	**Accuracy**	**City-Roadway**	**Expressway**	**Resting**	**Accuracy**
	**City-Roadway**	214	80	1	72.54%	87	50	2	62.59%
	**Expressway**	82	182	1	68.68%	29	59	1	66.29%
	**Resting**	2	0	31	93.94%	1	1	16	88.89%
**Actual**	**SVM Model**	**Training (Accuracy = 78.92%)**				**Testing (Accuracy = 68.70%)**			
		**City-Roadway**	**Expressway**	**Resting**	**Accuracy**	**City-Roadway**	**Expressway**	**Resting**	**Accuracy**
	**City-Roadway**	242	53	0	82.03%	96	42	1	69.06%
	**Expressway**	72	193	0	72.83%	33	56	0	62.92%
	**Resting**	0	0	33	100.00%	1	0	17	94.44%
**Actual**	**C5.0 Model**	**Training (Accuracy = 96.46%)**				**Testing (Accuracy = 64.63%)**			
		**City-Roadway**	**Expressway**	**Resting**	**Accuracy**	**City-Roadway**	**Expressway**	**Resting**	**Accuracy**
	**City-Roadway**	285	10	0	96.61%	82	56	1	58.99%
	**Expressway**	11	254	0	95.85%	27	61	1	68.54%
	**Resting**	0	0	33	100.00%	1	1	16	88.89%
**Actual**	**QUEST Model**	**Training (Accuracy = 67.45%)**				**Testing (Accuracy = 64.63%)**			
		**City-Roadway**	**Expressway**	**Resting**	**Accuracy**	**City-Roadway**	**Expressway**	**Resting**	**Accuracy**
	**City-Roadway**	217	76	2	73.56%	88	48	3	63.31%
	**Expressway**	108	151	6	56.98%	34	53	2	59.55%
	**Resting**	0	0	33	100%	0	0	18	100.00%

**Table 5:** Overall classification performance of machine-learning models for binary classification of EEG features of the resting and driving states. (a) The binary classification performance of EEG features of the resting and expressway driving states. (b) The binary classification performance of EEG features of the resting and the City-Roadway driving states. (c) The binary classification performance of EEG features of the City-Roadway driving and Expressway driving states.Overall performance parameters are the average of the training and testing classification performances.

(a) Model	Overall Accuracy	AUC	Gini
K-Nearest Neighbors Model	99.26%	1.000	0.999
C5.0 Model	99.26%	0.987	0.975
SVM Model	99.51%	1.000	1.000
Discriminant Analysis Model	99.26%	1.000	0.999
QUEST Model	97.53%	0.986	0.972
**(b) Model**	**Overall Accuracy**	**AUC**	**Gini**
K-Nearest Neighbors Model	98.97%	0.999	0.998
C5.0 Model	99.59%	0.999	0.997
SVM Model	99.79%	1.000	1.000
Discriminant Analysis Model	99.18%	0.999	0.999
QUEST Model	98.14%	0.990	0.979
**(c) Model**	**Overall Accuracy**	**AUC**	**Gini**
K-Nearest Neighbors Model	71.95%	0.779	0.559
C5.0 Model	75.38%	0.771	0.541
SVM Model	69.54%	0.77	0.541
Discriminant Analysis Model	68.27%	0.753	0.506
QUEST Model	65.48%	0.667	0.334

**Table 6:** Comparative study of methodologies and results between the current work and EEG-related studies.

Study	Study Sample	EEG Features	Findings	Application
Becker et al. [61]	40 subjects	EEG band powers, Phase Synchronization Index (PSI), Higher Order Crossing (HOC), Spectral Crest Factor (SCF), Fractal Dimension (FD)	Features extracted from the EEG band powers yield the best results, leading to overall classification scores of up to 70 or 75 percent.	Emotion Recognition Based on High-Resolution EEG
Iqram et al. [15]	37 stroke patients and 36 healthy elderly volunteers	EEG band powers, Revised Brain Symmetry Index, DAR, DTR	SVM model classified the stroke patients and the healthy adults with an accuracy of 92%	Disease prognostics using EEG
Halim et al. [62]	86 subjects	EEG band powers	SVM performs better to distinguish between rest and stress state with average classification accuracy of 97.95%	Driving-induced stress using EEG
Krishna et al. [63]	16 subjects	Alpha band powers	Mixture classification techniques classified different emotions with an average emotion recognition accuracy of 89%	Emotion Recognition Based on EEG
Proposed work	17 healthy adult drivers	Alpha band powers	EEG delta, theta, DAR, DTR features showed stronger correlations with driving states.	Driving-induced mental workload using EEG

## Supplementary Materials

The following are available online at https://www.mdpi.com/article/10.3390/s21216985/s1, Table S1a: Overall Classification Performance of Machine-learning model while repeated k-fold cross validation for binary classification of EEG features of the resting and Expressway driving states. Table S1b: Overall Classification Performance of Machine-learning model while repeated k-fold cross validation for binary for classification of EEG features of the resting and the City-Roadway driving states. Table S1c: Overall Classification Performance of Machine-learning model while repeated k-fold cross validation for binary classification of EEG features of the City-Roadway driving and Expressway driving states. Figure S1. Frontal EEG spectral power asymmetry features during resting-state and driving along the City-Roadway, and Expressway. (a) Frontal alpha asymmetry for resting and driving states. (b) Frontal beta asymmetry for resting and driving states. (c) Frontal theta asymmetry for resting and all driving states (*p* < 0.05). (d) Frontal delta asymmetry for resting and driving states. (e) Frontal gamma asymmetry for resting and driving states. * indicates *p* < 0.05.
