# Supplementary material for: Driving-Induced Neurological Biomarkers in an Advanced Driver-Assistance System
Source: Sensors (Basel). 2021 Oct 21;21(21):6985. doi: 10.3390/s21216985 (PMC8588463; doi:10.3390/s21216985)
Supplement: Supplementary file 1 [file sensors-21-06985-s001.zip › sensors-1393092-supplementary.pdf]

sensors

Hussain, Iqram, Young, Seo, Park, Se Jin. Driving-induced Neurological Biomarkers in an Advanced Driver-Assistance System. *Sensors*. **2021**, *21*, 6985.

Table S1a. Overall Classification Performance of Machine-learning model while repeated k-fold cross validation for binary classification of EEG features of the resting and Expressway driving states. Overall performance parameters are the average of the training and testing classification performances.

Model	Overall Accuracy (Repeated k-fold cross validation)		
	Repeat_1	Repeat _2	Repeat_3
K-Nearest Neighbors Model	99.39%	98.59%	99.52%
C5.0 Model	99.18%	99.26%	98.93%
SVM Model	98.79%	99.51%	98.79%
Discriminant Analysis Model	99.26%	99.26%	99.59%
QUEST Model	97.53%	98.39%	98.13%

Table S1b. Overall Classification Performance of Machine-learning model while repeated k-fold cross validation for binary for classification of EEG features of the resting and the City-Roadway driving states. Overall performance parameters are the average of the training and testing classification performances.

Model	Overall Accuracy (Repeated k-fold cross validation)		
	Repeat_1	Repeat _2	Repeat_3
K-Nearest Neighbors Model	99.13%	97.63%	99.13%
C5.0 Model	99.26%	98.26%	99.63%
SVM Model	98.93%	99.39%	98.49%
Discriminant Analysis Model	98.63%	98.93%	99.26%
QUEST Model	98.59%	99.59%	98.63%

Table S1c. Overall Classification Performance of Machine-learning model while repeated k-fold cross validation for binary classification of EEG features of the City-Roadway driving and Expressway driving states. Overall performance parameters are the average of the training and testing classification performances.

Model	Overall Accuracy (Repeated k-fold cross validation)		
	Repeat_1	Repeat _2	Repeat_3
K-Nearest Neighbors Model	70.63%	71.43%	72.39%
C5.0 Model	73.93%	74.53%	76.13%
SVM Model	72.19%	70.63%	69.18%
Discriminant Analysis Model	69.73%	69.39%	71.43%
QUEST Model	68.39%	67.93%	66.23%

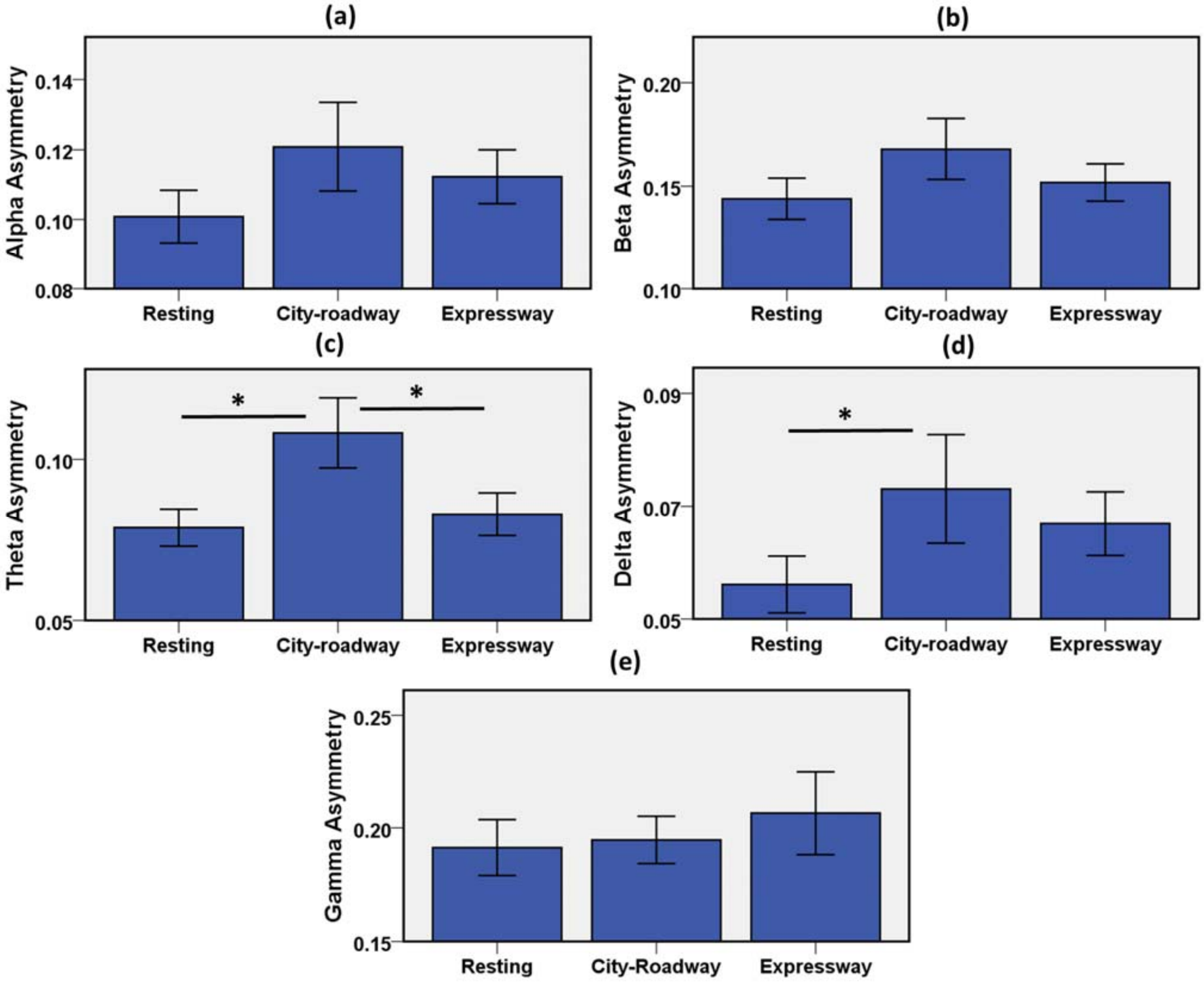

Figure S1. Frontal EEG spectral power asymmetry features during resting-state and driving along the City-Roadway, and Expressway. (a) Frontal alpha asymmetry for resting and driving states. (b) Frontal beta asymmetry for resting and driving states. (c) Frontal theta asymmetry for resting and all driving states ($p < 0.05$). (d) Frontal delta asymmetry for resting and driving states. (e) Frontal gamma asymmetry for resting and driving states. * indicates $p < 0.05$.
